# What can food-image tasks teach us about anorexia nervosa? A systematic review

**DOI:** 10.1186/s40337-018-0217-z

**Published:** 2018-11-01

**Authors:** E. Caitlin Lloyd, Joanna E. Steinglass

**Affiliations:** 1 Centre for Exercise, Nutrition and Health Sciences, School for Policy Studies, University of Bristol, Bristol, UK; 2 Department of Psychiatry, Columbia University Irving Medical Center, New York, NY USA; 3 New York State Psychiatric Institute, New York, NY USA

**Keywords:** Anorexia nervosa, Eating behaviour, Food stimuli, Eating disorders, Cognitive neuroscience, fMRI

## Abstract

A salient feature of anorexia nervosa (AN) is the persistent and severe restriction of food, such that dietary intake is inadequate to maintain a healthy body weight. Experimental tasks and paradigms have used illness-relevant stimuli, namely food images, to study the eating-specific neurocognitive mechanisms that promote food avoidance. This systematic review, completed in accordance with PRISMA guidelines, identified and critically evaluated paradigms involving images of food that have been used to study AN. There were 50 eligible studies, published before March 10^th^ 2018, identified from Medline and PsychINFO searches, and reference screening. Studies using food image-based paradigms were categorised into three methodologic approaches: neuropsychology, neurophysiology, and functional magnetic resonance imaging (fMRI). Paradigms were reviewed with a focus on how well they address phenomena central to AN. Across tasks, differences between individuals with AN and healthy peers have been identified, with the most consistent findings in the area of reward processing. Measuring task performance alongside actual eating behaviour, and using experimental manipulations to probe causality, may advance understanding of the mechanisms of illness in AN.

## Plain English Summary

A core feature of anorexia nervosa is the persistent limiting of calorie intake, or avoidance of eating, which results in a severely low body weight. Given the relevance of eating behaviour to anorexia nervosa, studies have often used tasks involving pictures of food to try and understand the factors that contribute to the illness. In this article, we review studies involving the presentation of food images, to highlight the approaches that have been most successful in furthering knowledge about AN. Studies to date have identified some differences among individuals with AN, but have had limited success in identifying underlying mechanisms of illness. We consider modifications to existing experimental designs that may address these limitations, in particular discussing methods that have been used to study eating behaviour in non-eating disorder populations. We conclude that when using food image tasks to develop a better understanding of anorexia nervosa, it is important to link actual eating behaviour to task outcomes, and to develop research based on more specific hypotheses.

## Background

A core feature of anorexia nervosa (AN) is disturbed eating behaviour [1]. Individuals with AN limit overall caloric intake and specifically calories derived from fat. This pattern of eating persists following weight recovery, and is associated with relapse in the longer term [2, 3]. AN is a complex illness, and there are generalized neurocognitive deficits (e.g., set shifting, central coherence) [4, 5]. Yet, maladaptive eating behavior is central to the definition of AN, leading to models and proposed neurobiological mechanisms of illness centered around aberrant brain responses to food [6]. Measures of neuropsychological processes, such as the Stroop task [7], have been adapted to include illness-specific words, which is thought to increase the attentional deficits among patients [8]. To understand maladaptive eating behaviour, experimental tasks have utilized images of food.

The primary purpose of this review is to identify and critically evaluate the food image *tasks and paradigms* that have been used in the study of AN. This systematic literature review identified neuropsychological, neurophysiological, and neuroimaging studies. Across these approaches, task domains included attention, reward, perception, and decision-making. While study findings are discussed, we primarily provide a qualitative review of the tasks themselves, and discuss how well these paradigms address phenomena central to AN.

## Methods

### Search strategy and eligibility criteria

**Fig. 1:**
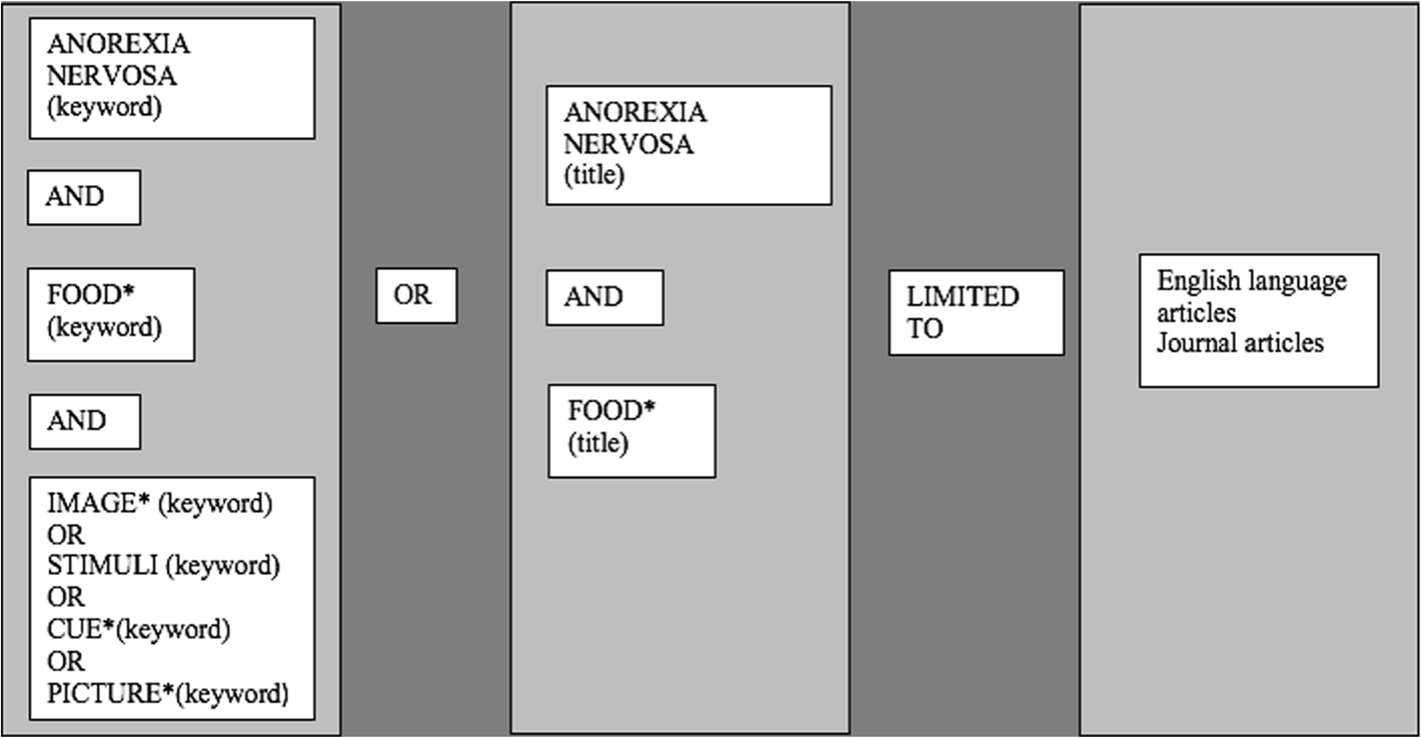
Systematic Review Search Strategy

PsychInfo and Medline were searched, using the search strategy shown in Fig. 1. Following deduplication, titles and abstracts were screened to determine whether the article should be included in the full-text screening stage. References in eligible studies were manually screened to identify studies not captured by database searches. Table 1 details inclusion criteria.

**Table 1:** Systematic Review Inclusion Criteria

Sample population	Study included a defined group of individuals with AN or atypical AN (acutely ill or weight-restored). AN group was analysed separately (i.e., studies with only mixed ED analyses were not included)
Study design	Administration of a paradigm involving the presentation of real food images (i.e., not cartoons)
Study type	Primary investigations only, secondary data analyses were not included
Outcome measure	Any
Date of study	Up to and including 10th March 2018
Publication type	Peer-reviewed full-text journal articles
Language	English

### Data Extraction, Synthesis, and Quality Assessment

Participant details (age, AN subtype, BMI), task information (nature of task, observed variables) and study information (sample size, use of non-food control images, standardisation of pre-task intake, findings) were extracted. Studies were categorized by type (neuropsychology, neurophysiology, neuroimaging). The neuropsychology and neurophysiology tasks were further categorised by the following neurocognitive domains: attention, reward, perception, and decision-making. The qualitative review identified strengths and limitations of the tasks.

We used the Ferro and Speechley version [9] of the Downs and Black Quality Index [10] to provide a systematic assessment of study quality. A number of items were not applicable to the current review given the neurobiological nature of included studies. Consistent with an existing review [11], we removed the following items: response rate; estimates of the random variability; staff / places / facilities where patients studied representative; outcome measures valid and reliable. We also removed the confounding item given potential confounders varied substantially across studies. The final index consisted of 10 items; 10 was the maximum score (quality index item-level results available upon request).

## Results

**Fig. 2:**
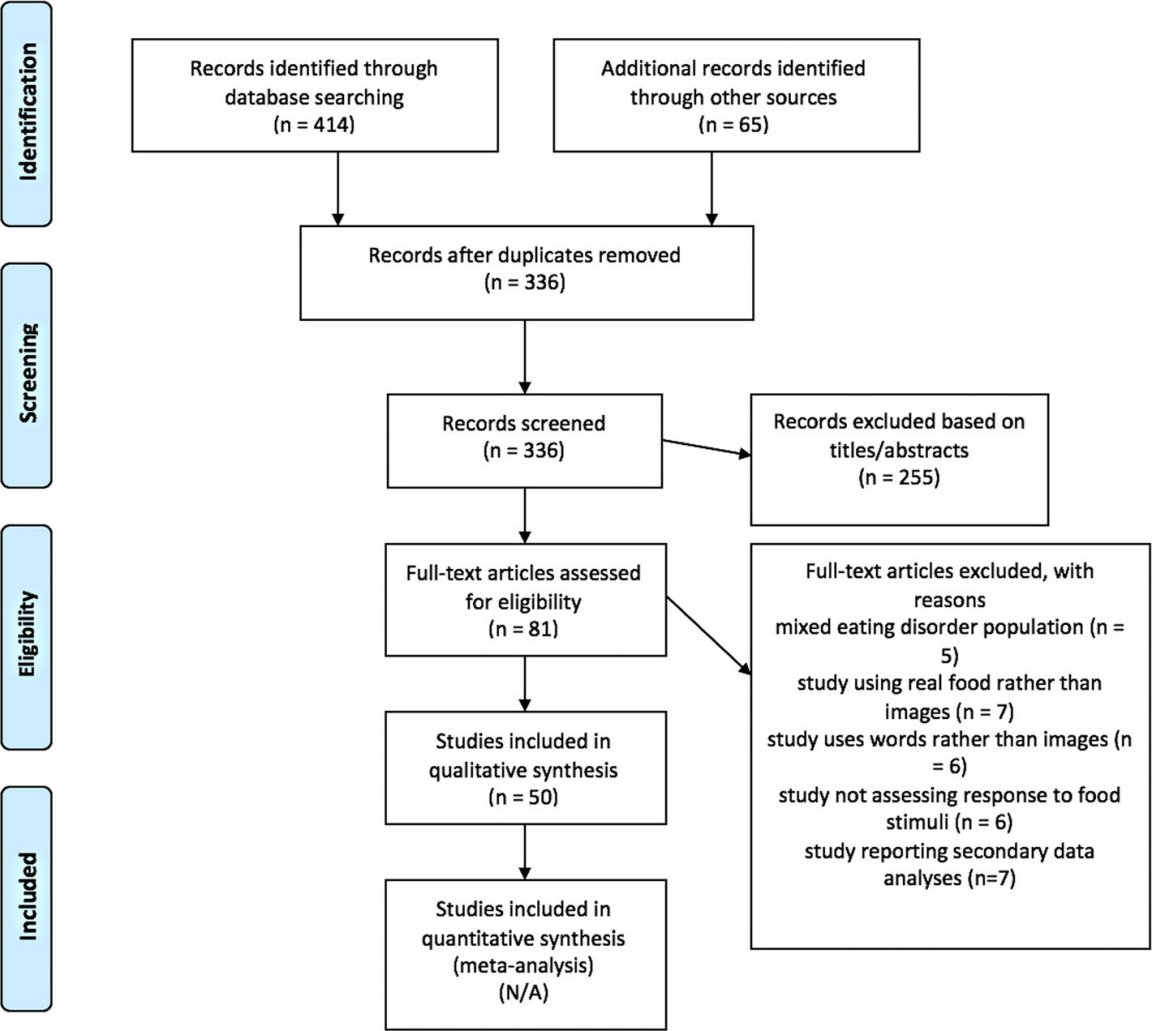
PRISMA Flow Diagram

We identified 50 studies meeting eligibility criteria (Figure 2, PRISMA flow diagram). These studies are presented in Table 2.

**Table 2:** Food-image tasks in the study of anorexia nervosa

Domain	Subdomain	Study	Paradigm & Outcome Variable(s)	Participants (AN subtype)	Mean age (SD)	Mean BMI (SD)	Non-food images presented	Meal-Task Interval Standardised	Summary of Findings	Quality Index Score
Neuropsychology: Attention	Distraction	Dickson et al., 2008 [19]	Working memory task. Effects of image presentation on reaction time and accuracy.	24 AN (R)	30.6 (9.0)	16.0 (1.0)	Yes	No	No differences AN vs HC	6
				24 HC	33.1 (8.0)	21.9 (2.0)				
		Brooks et al., 2012 [20]	Working memory and inhibitory control task. Effects of image presentation on reaction time and accuracy.	13 AN (R)	25 (11.0)	15 (1.54)	Yes	Yes	Distraction during working memory task: AN > HC Distraction during inhibitory control task: AN < HC	7
				20 HC	22 (5.0)	22.38 (2.66)				
		Neimeijer et al., 2017 [18]	Visual target detection task. Effects of image presentation on accuracy.	66 AN (57.5% R, 42.5% EDNOS AN-R)	15.25 (1.86)	15.45 (1.79)	Yes	No	Distraction AN > HC	6
				55 HC	16.14 (1.9)	20.45 (2.10)				
	Recognition	Nikendei et al., 2008 [62]	Participants view images. Recognition and recall.	16 AN (81.25% R, 18.75% BP)	22.8 (U)	14.8 (2.3)	Yes	No	No differences AN vs HC	9
				16 HC, fasted	23.6 (U)	U				
				16 HC, sated	23.1 (U)	U				
	Visual probe detection	Veenstra & de Jong 2012 [24]	Exogenous cueing task: participants detect targets that appear in one of two on-screen locations; distractor image (food or neutral stimuli) precedes target onset, appearing in either the same (valid trials) or opposite (invalid trials) location as the target. Response time.	88 AN (61.3% R, 28.7% EDNOS AN-R)	15.02 (1.37)	15.69 (1.90)	Yes	No	No differences AN vs HC	6
				76 HC	15.12 (1.75)	20.42 (2.37)				
		Cardi et al., 2012 [28]	Dot-probe task: Participants instructed to detect targets appearing in one of two on-screen locations, either replacing a food or neutral image that are presented concurrently. Response time.	18 AN (33% R, 11% BP, 55% EDNOS-AN)	31.5 (11.4)	17 (2.6)	Yes	No	No effect of intervention on attentional bias towards food	5
				19 HC	28.6 (8.0)	23.9 (2.8)				
		Cardi et al., 2013 [27]		38 AN (21% EDNOS)	29.9 (7.92)	16.2 (2.35)	Yes	No	Attentional bias towards food inpatients: pre-meal < post-meal Attentional bias towards food outpatients: pre-meal > post-meal	6
		Kim et al., 2014 [23]		31 AN	23.1 (9.35)	15.15 (2.51)	Yes	Yes	No differences AN vs HC	7
				33 HC	22.18 (2.14)	20.91 (2.22)				
		Cardi et al., 2015 [29]		19 AN	31.0 (10.0)	16.7 (2.7)	Yes	Yes	No differences AN vs HC	7
				23 BN	24.4 (5.7)	23.4 (6.9)				
				36 HC	25.9 (5.0)	21.5 (2.0)				
		Leppenan et al., 2017 [26]		30 AN	26.2 (6.82)	16.3 (2.04)	Yes	Yes	Attentional bias away from food: AN > HC	8
				29 HC	26.83 (8.54)	23.25 (3.65)				
	Eye-tracking	Giel et al., 2011 [32]	Eye movements recorded while participants view pairs consisting of a food and neutral image. Number and duration of fixations on images.	19 AN (73.6% R, 26.4% BP)	24.4 (4.1)	15.8 (1.8)	Yes	Yes	Attentional bias towards food: AN < HC	7
				20 HC, sated	24.2 (2.9)	21.3 (1.7)				
				18 HC, fasted	24.4 (2.6)	21.6 (1.5)				
Neuropsychology: Reward	Explicit rating	Bossert et al., 1991 [53]	Participants view images. Palatability ratings.	9 AN (R)	21.9 (2.9)	66.1(8.5) IBW	No	No	Palatability high calorie food: AN < HC Palatability low calorie food: AN > HC	8
				20 BN	21.7 (2.9)	101.9 (12.9) IBW				
				9 HC	22.3 (1.2)	100.8 (4.8) IBW				
		Jiang et al., 2010 [46]	Participants view images. Liking and wanting ratings.	17 AN (R)	26.47 (7.12)	15.04 (1.93)	Yes	Yes	Liking: AN < HC Wanting: AN < HC	6
				29 HC	24.52 (5.58)	20.38 (1.87)				
		Krizbai et al., 2016 [52]	Participants view images. Valence, dominance, arousal ratings.	14 AN (R)	15.07 (1.38)	16.92 (1.73)	No	No	Positive valence: AN < HC	5
				14 HC	15.14 (1.29)	20.51 (2.50)				
	Implicit rating	Cowdrey et al., 2013 [41]	Binary forced choice procedure: across multiple trials participants choose between two foods, each of which may be high or low calorie and sweet or savoury. Relative response time to select high and low-calorie foods (implicit wanting).	20 AN (80% R, 20% BP)	26.4 (10.56)	16.33 (1.1)	No	No	Explicit wanting high calorie food: AN < HC; AN-WR < HC Implicit wanting high calorie food: AN < HC; AN < AN-rec; AN-WR < HC Implicit wanting low calorie food: AN > HC; AN > AN-rec; AN-WR > HC	4
				22 AN-WR (82% R, 18% BP)	25.1 (6.03)	21.05 (1.89)				
				22 AN-rec (82% R, 18% BP)	23.73 (5.76)	21.03 (1.53)				
				41 HC	24.29 (6.46)	21.7 (1.88)				
	Approach-Avoidance	Spring & Bulik, 2014 [51]	Affect misattribution procedure: participants briefly view food images, which are replaced by Chinese characters. Pleasantness ratings of Chinese characters (implicit liking).	9 AN	21.4 (5.79)	U	Yes	No	Implicit liking: AN < HC	6
				14 AN-rec						
				29 HC						
		Veenstra & de Jong, 2011 [54]	Manikin Task: participants move manikin towards or away from images presented on-screen depending on orientation of image (horizontal or vertical). Errors/reaction times on approach versus avoidance trials (approach bias).	89 AN (60.7% R, 39.3% EDNOS AN-R)	14.84 (1.70)	15.71 (1.87)	Yes	No	Approach bias: AN < HC	6
				76 HC	14.86 (1.70)	20.42 (2.37)				
		Neimeijer et al., 2015 [56]		98 AN (64.5% AN, 45.5% EDNOS-AN)	14.97 (1.63)	U	Yes	No	Approach bias high calorie food: pre-treatment < post-treatment	8
		Paslaskis et al., 2016 [55]	Approach-avoidance task: participants push or pull computer mouse depending on orientation of on-screen image (horizontal or vertical). Reaction time on approach versus avoidance trials (approach bias).	41 AN (80.5% R, 19.5% BP)	26.85 (6.71)	15.29 (1.6)	No	Yes	Approach bias: AN < HC	6
				42 HC	24.79 (2.71)	21.37 (1.57)				
Neuropsychology: Perceptual tasks	Size perception	Yellowlees et al., 1988 [57]	Participants view real food item and adjust screen image to match perceived size. Estimated versus actual size of items.	20 AN	22.4 (8.0)	U	Yes	No	Size over-estimation: AN > HC	5
				20 HC	22.0 (6.6)					
		Milos et al., 2013 [58]	Participants view meals of different portion sizes. Estimates of portion size.	24 AN	22.38 (4.10)	15.8 (2.01)	No	Yes	Portion size estimate: AN > HC	7
				27 HC	21.41 (2.75)	21.47 (2.71)				
		Kissileff et al., 2016 [99]	Participants view meals of different portion sizes. Tolerability and expected anxiety ratings.	24 AN (87.5% R, 22.5% BP)	15.46 (1.57)	17.11 (1.35)	No	No	Portion size tolerability: AN < HC Anticipated anxiety: AN > HC	7
				10 HC	14.6 (2.63)	20.6 (1.35)				
	Weight-gain estimate	Milos et al., 2017 [100]	Participants view meals of different portion sizes. Estimates of weight-gain as a consequence of eating portions presented.	24 AN	22.38 (4.10)	15.8 (2.01)	No	Yes	Estimation of weight-gain: AN > HC	7
				27 HC	21.41 (2.75)	21.47 (2.71)				
Neuropsychology: Decision making	Food-choice	Steinglass et al., 2015 [60]	Participants select between food items varying in fat content in a binary forced choice task. Proportion of high fat food items selected.	22 AN (54.5% R, 45.5% BP)	29.4 (11.2)	17.5 (1.9)	No	No	Preference for high-fat food items: AN < HC	6
				22 HC	26.3 (5.8)	21.0 (1.7)				
Neurophysiology: Attention	Electroencephalography	Blechert et al., 2011 [67]	Neural activity recorded with electroencephalography (EEG) while food images viewed. Amplitude of neural response.	21 AN	23.2 (4.55)	16.6 (1.3)	Yes	No	Neuronal activity: AN > HC	7
				22 BN	26.1 (7.5)	22.6 (3.24)				
				32 HC	26.2 (5.02)	20.7 (2.41)				
		Nikendei et al., 2012 [69]		16 AN (81.25% R, 18.25% BP)	22.8 (5.2)	14.8 (2.3)	Yes	No	No differences AN vs HC	8
				16 HC pre-meal	23.1 (4.8)	22.3 (2.1)				
				16 HC post-meal	23.6 (5.2)	20.9 (1.7)				
		Novosel et al., 2014 [68]		11 AN	15.36 (1.62)	15.79 (1.87)	Yes	No	Neuronal activity: AN > HC	6
				11 HC		20.42 (1.77)				
	Magnetoencephalography	Godier et al., 2016 [33]	Neural activity recorded with magnetoencephalography (MEG) while participants think about how much they want to eat presented food images. Amplitude of neural response.	13 AN (R)	31.2 (5.3)	15.7 (1.9)	Yes	Yes	Neuronal activity at 150 ms: AN > HC; AN > AN-rec Neuronal activity at 320 ms: AN < AN-rec	6
				14 AN-rec (R)	27.1 (6.5)	20.9 (1.6)				
				15 HC	23.7 (5.4)	21.4 (1.9)				
Neurophysiology: Reward	Electromyography	Soussignan et al., 2010 [71]	Participants view food images presented following subliminal presentation of facial expressions. Activity of facial (zygomatic, corrugator) muscles index hedonic response.	16 AN (R)	26.68 (7.30)	14.97 (1.97)	No	Yes	Corrugator activity: AN > HC Zygomatic activity: AN < HC	7
				25 HC	24.6 (6.03)	20.52 (1.90)				
		Soussignan et al., 2011 [72]	Participants view food images. Activity of facial (zygomatic, corrugator) muscles index hedonic response.	17 AN (R)	26.5 (7.1)	14.9 (1.9)	Yes	Yes	Corrugator activity: AN > HC	6
				27 HC	24.7 (6.1)	20.4 (1.8)				
		Hildebrandt et al., 2015 [73]	Participants complete food-based associative learning task. Activity of facial muscles (zygomatic, corrugator, levator labii), index hedonic and disgust responses.	14 AN/EDNOS-AN (R)	15.05 (1.87)	17.52 (2.91)	No	No	Levator labii activity to food cue: AN > HC Zygomatic activity to cue for absence of food: AN > HC	5
				15 HC	17.64 (2.71)	22.49 (2.94)				
		Friederich et al., 2006 [74]	Acoustic stimulus and food image presented concurrently; activity of oculomotor muscle recorded. Startle response indexes appetitive/aversive motivation.	13 AN (84.6% R, 25.4% BP)	25.1 (3.7)	16.4 (3.7)	Yes	Yes	No differences AN vs HC	7
				15 BN	25.2 (5.1)	23.4 (3.7)				
				25 HC	25.0 (3.3)	21.8 (2.7)				
		Racine et al., 2016 [76]		19 AN (36.8% R, 63.2% BP)	25.11 (9.13)	16.72 (1.63)	Yes	No	AN unable to suppress startle response to food and negative non-food stimuli	7
		Erdur et al., 2017 [75]		33 AN (60.6% R, 39.4% BP)	28.2 (9.41)	15.8 (1.90)	Yes	Yes	No differences AN vs AN-rec vs HC	7
				15 AN-rec (33.3% R, 66.6% BP)	40.8 (6.79)	20.81 (1.93)				
				18 HC	28.95 (8.25)	21.82 (1.58)				
Neuroimaging	Passive viewing	Nagamitsu et al., 2012 [82]	Participants view images of food stimuli while brain activity measured with functional near infrared spectroscopy. Neural response.	12 AN (R)	14.4 (1.3)	15.5 (2.0)	Yes	No	No differences AN vs HC	5
				13 HC	14.3 (1.3)	18.7 (1.3)				
		Ellison et al., 1998 [78]	Participants view images of food stimuli while brain activity measured with fMRI. Neural response.	6 AN	U	15	No	No	Brain activation: AN > HC (insula, anterior cingulate gyrus, amygdala)	6
				6 HC		U				
		Santel et al., 2006 [48]		13 AN (R)	16.1 (2.0)	16.0 (1.7)	Yes	Yes	Brain activation pre-meal: AN < HC (lingual gyrus); Brain activation post-meal: AN < HC (Inferior parietal lobule)	6
				10 HC	16.8 (2.6)	20.5 (1.9)				
		Gizewski et al., 2010 [47]		12 AN (R)	27 (U)	14.1 (1.8)	Yes	Yes	Brain activation pre-meal: AN > HC (midcingulate cortex); AN < HC (ACC) Brain activation post-meal: AN > HC (L insula); HC > AN (prefrontal cortex and R insula)	6
				10 HC	25 (U)	21.4 (1.5)				
		Joos et al., 2011 [44]		11 AN (R)	25.0 (5.0)	16.2 (1.2)	Yes	No	Brain activation: AN > HC (amygdala); AN < HC (midcingulum)	6
				11 HC	26.0 (5.2)	21.1 (1.8)				
		Rothemund et al., 2011 [79]		12 AN (83.4% R, 16.6% BP)	24 (6.1)	13.6 (1.2)	Yes	Yes	Brain activation: AN > HC (precuneus)	5
				12 HC	26 (3.7)	21 (1.6)				
		Holsen et al., 2012 [45]		12 AN (R)	21.8 (2.7)	18.0 (0.8)	Yes	Yes	Brain activation pre-meal: AN < HC (amygdala, hypothalamus, insula, hippocampus, orbitofrontal cortex); AN-WR < HC (hypothalamus, amygdala, insula) Brain activation post-meal: AN < HC (insula, amygdala); AN > AN-WR (amygdala); AN < AN-WR (insula)	6
				10 AN-WR	23.4 (2.3)	22.1 (2.2)				
				11 HC	21.6 (1.3)	22.4 (1.3)				
		Kim et al., 2012 [84]		18 AN (33.3% R, 66.6% BP)	25.2 (4.2)	16.0 (3.7)	Yes	Yes	Brain activation: AN > HC (Inferior frontal gyrus, anterior cingulate cortex, superior frontal gyrus and cerebellem); AN > BN (anterior cingulate cortex); AN < BN (middle temporal gyrus)	6
				20 BN	22.9 (3.9)	21.6 (2.3)				
				20 HC	23.3 (1.8)	19.9 (1.9)				
		Boehm et al., 2017 [83]		35 AN (94.2% R, 5.8% BP)	16.25 (3.46)	14.59 (1.5)	Yes	No	Brain activation: AN > HC (superior occipital gyrus)	7
				25 HC	16.31 (3.39)	20.46 (2.06)				
		Kerr et al., 2017 [80]		20 AN-WR (R)	17 (3)	18 (3)	Yes	No	Brain activation correlates with interoceptive awareness in opposite directions AN vs HC	5
				20 HC	19.84 (0.87)	21.3 (1.55)				
	Directed food tasks	Uher et al., 2003 [85]	Participants shown images of food while neural activity measured with fMRI; participants instructed to think how hungry images make them feel and whether they would like to eat the food. Neural response.	8 AN (R)	25.6 (2.8)	16.6 (1.2)	Yes	Yes	Brain activation: AN < AN-rec (Apical prefrontal cortex, dorsolateral prefrontal cortex, medial paracentral cortex, cerebellem); AN > AN-rec (occipital-lingual gyrus)	5
				9 AN-rec (R)	26.9 (5.3)	20.4 (2.1)				
				9 HC	26.6 (3.3)	22.2 (3.8)				
		Uher et al., 2004 [50]		16 AN (56.8% R, 43.2% BP)	26.93 (12.14)	16.04 (1.64)	Yes	Yes	Brain activation: AN > HC (medial orbitofrontal cortex, lingual gyrus, anterior cingulate); AN < HC (parietal cortex, cerebellem); AN > BN (apical prefrontal cortex, lateral prefrontal cortex, occipital lingual gyrus); AN < BN (cerebellem)	6
				10 BN	29.80 (8.80)	22.43 (2.37)				
				19 HC	26.68 (8.34)	22.41 (2.98)				
		Brooks et al., 2011 [81]	Participants shown images of food while neural activity measured with fMRI; participants instructed to imagine eating the food. Neural response.	18 AN (61.1% R, 38.9% BP)	26.0 (6.8)	U	Yes	Yes	Brain activation: AN > BN (parietal lobe, cingulate cortex); AN < BN (superior temporal gyrus, caudate, supplementary motor area); AN > HC (visual cortex); AN < HC (cerebellem)	7
				8 BN	25.0 (7.1)					
				24 HC	26.0 (9.5)					
		Sanders et al., 2015 [49]		15 AN (60% R, 40% BP)	25.6 (5)	14.5 (1.7)	Yes	Yes	Brain activation: AN < HC (superior frontal gyrus); AN > HC (middle frontal gyrus)	6
				15 AN-rec (66.6% R, 33.3% BP)	24.3 (5)	21.1 (1.9)				
				15 HC	25.8 (5)	21.5 (2.3)				
		Scaife et al., 2016 [43]	Participants shown images of food while neural activity measured with fMRI; participants instructed to think about how much they want to eat the food at the present moment. Neural response.	12 AN (R)	29.4 (6)	15.4 (1.9)	No	Yes	Brain activation: AN < HC (postcentral gyrus, precuneus, superior parietal lobule); AN < HC (frontal pole, dorsolateral prefrontal cortex, supramarginal gyrus for low calorie food); AN > HC (frontal pole for high calorie food)	6
				14 AN-rec (R)	27 (6.5)	20.9 (1.6)				
				16 HC	24.3 (5.7)	21.2 (2)				
	Active choice	Foerde et al., 2015 [61]	Participants select between food items varying in fat content in a binary forced choice task; fMRI measures neural activity. Neural activity during food choice.	21 AN (47.6% R, 52.4% BP)	26.1 (6.5)	21.5 (1.9)	No	Yes	Brain activation: AN > HC (dorsal striatum)	6
				21 HC	22.7 (3.1)	15.7 (2.0)				

*AN* individuals with AN, *AN-rec* individuals recovered from AN, *AN-WR* weight recovered individuals with AN, *BP* binge-purge subtype, *EDNOS* Individuals with eating disorder not otherwise specified, *HC* healthy controls, *IBW* ideal body weight, *R* restricting subtype, *U* unreported

### Neuropsychology

#### Attention

Attention directs sensory and cognitive processing, which in turn influences behaviour [12]. An attentional bias refers to the differential allocation of attention towards one set of stimuli over another. There are three components of attentional bias: increased capture of attention by a stimulus (orienting); reduced ability to direct attention away from a stimulus once attention has been captured (disengagement); and efforts to avoid allocating attention to a stimulus (attentional avoidance) [13]. Components of attentional bias have been studied in other areas of psychopathology, such as anxiety disorders. Individuals with anxiety disorders demonstrate an enhanced orienting towards “threat-related” stimuli, along with difficulties disengaging from such stimuli [13]. These biases are related to anxiety severity [14, 15]. It has been hypothesised that individuals with AN might show similar attentional biases towards food stimuli, indicating a perceived threat, that might in turn be associated with greater dietary restriction [16]. On the other hand, attention to a food stimulus predicts consumption of that food (i.e., the opposite of dietary restriction [17]) in healthy individuals. As such, an alternative hypothesis is that individuals with AN would show *reduced* attentional bias towards food, and that this is a mechanism that facilitates dietary restriction [18]. Attention paradigms incorporating pictures of food have been administered to individuals with AN to test these competing hypotheses, to assess whether altered attention to food underlies maladaptive dietary restriction.

In distraction paradigms (Table 2, Distraction) pictures of food are presented while participants engage in an unrelated cognitive task. The difference in task performance when food images are presented as distractor items (as compared to neutral distractors) indicates an attentional bias. Individuals with AN were less accurate than healthy controls (HC) in an attention task when distracted by food-related stimuli, with no group difference in the presence of neutral stimuli [18]. One study reported no differential effect of food versus other distractors on working memory accuracy among individuals with AN or HC [19]. However, another study reported working memory performance was compromised by food stimuli distractors among AN, but not HC [20]. In an inhibitory control task, food-image presentation was associated with decreased accuracy among HC, but not AN [20].

In visual probe detection tasks [21, 22] participants identify the location of visual probes that replace food and non-food images (Table 2, Visual probe detection). Faster responses on food trials indicate greater attention to food stimuli (attentional bias towards food). Three studies found no evidence of differences between AN and HC in attentional bias towards food [23–25]. A fourth study found that when fasted, individuals with AN, compared with HC, displayed an attentional bias away from food stimuli [26]. Two studies of the effect of oxytocin [23, 26] and three of mealtime support interventions [27–29] explored whether attentional bias towards food mediated intervention effects on intake. Changes in attentional bias tended not to correspond with changes in food intake in AN, although small sample sizes limited detection of intervention effects in these studies.

Eye-tracking devices measure attention by monitoring the frequency and duration of gaze fixations at particular positions on a computer screen (Table 2, Eye-tracking [30, 31]). One study found AN and HC did not differ in initial fixation points when high-calorie food and household items were presented concurrently, but the duration of fixations on food-images was shorter for individuals with AN [32]. When single images of food were presented, eye-gaze measurements did not differ between AN and HC [33].

There do not appear to be robust differences in attention bias to food between AN and HC, though some studies of distraction suggest group differences. Interestingly, attention may be differentially associated with intake in the two populations.

#### Reward

Food is generally considered a primary reward [34], and therefore is useful for understanding how reward is assessed and processed. One approach to studying reward distinguishes separate components of liking and wanting [35, 36]. ‘Liking’ refers to the hedonic impact, or pleasure; ‘wanting’ refers to the motivation to consume a reward [37]. The rewarding properties of a food influence consumption among HC [38, 39]. This observation has resulted in the hypothesis that individuals with AN, who consume less food, do not experience food as rewarding [40], or that ‘liking’ or ‘wanting’ do not drive food consumption in the expected ways [41, 42]. To examine this, tasks have probed the reward value and processing of food by presenting images of food.

In the most straightforward behavioural paradigms, participants rate how pleasurable they find images of food, and/or how much they want to eat those foods (Table 2, Explicit rating task). Across multiple studies (behavioural and neuroimaging), individuals with AN report liking and wanting food less than HC, especially high-calorie foods [33, 41, 43–53]. A variation of this task asks individuals to rate neutral images presented immediately after food stimuli (Table 2, Implicit rating task). Neutral images that follow food images are rated as less pleasant by AN than HC [51].

In forced-choice tasks, speed of response provides a behavioural index of motivation, separate from subjective ratings (Table 2, Binary forced choice task). For example, an individual may report not wanting to eat certain foods, but then respond rapidly to these foods during a task. This rapid response is thought to indicate an implicit ‘wanting’ that differs from the explicit rating. Across two studies, individuals with AN were slower than HC when selecting between two high-calorie foods [41, 43], and explicit wanting scores were also lower. In one study (but not the other) individuals with AN were faster than HC to select low-calorie foods [41]; this indication of greater implicit wanting was not reflected in the explicit response.

In approach-avoidance paradigms, participants are presented with images of food and neutral items, and instructed to either move toward (approach) or away from (avoid) the image, according to image orientation (i.e., portrait vs landscape). Approach-avoidance paradigms are based on the assumption that it is easier to approach than to avoid stimuli with motivational value. Error rates and response latencies on approach trials are subtracted from avoidance trials to calculate bias (positive values reflect an approach bias). Scores in the neutral condition are subtracted from the food condition, to calculate food-specific approach/avoidance bias. One study found that individuals with AN made fewer errors than HC on avoidance food trials, resulting in a reduced approach bias to food stimuli [54]. Another study found smaller differences in response times on food approach versus avoidance trials among individuals with AN, also yielding a reduced approach bias in AN [55]. Adolescents with AN displayed an approach bias toward low-calorie, but not high-calorie, food stimuli before treatment [56]. After treatment, an approach bias was present for both types of food. These results are difficult to interpret in the absence of a HC group; one optimistic interpretation is that ‘wanting’ for high-calorie food increased with treatment.

The collection of findings from reward processing tasks suggest decreased value or reward of high-calorie food among individuals with AN, with mixed findings for low-calorie stimuli.

#### Perception

Individuals with AN often over-report caloric intake, despite under-consumption of food, raising a question about whether patients have a perceptual deficit. Some tasks test this idea by assessing perception of images of food and portion sizes (Table 2; Perceptual tasks).

In one task, individuals adjust the size of images of food and neutral items presented on screen until these are perceived to match the size of actual items. Individuals with AN over-estimated food sizes relative to HC, while estimates of neutral object size were similar between groups [57]. When presented with portions of food, individuals with AN overestimated the size of smaller (but not larger) portions [58]. Individuals with AN, but not HC, reported meal size to be larger when imagining they would eat the meal (intent to eat condition) compared to considering meal size “in general” (general condition).

These studies identified differences in assessments of portion size between AN and HC, with no overall perceptual deficits.

#### Decision-Making

The only food-image decision-making task administered in AN measures food-choice, or, how individuals make decisions about what to eat [59, 60]. In this task, participants rate a series of images of food for both healthiness and tastiness. One item that was rated neutral for both healthiness and tastiness is selected to be a “reference item” and participants then choose between the reference item and each of the other foods (Table 2, Decision-making). In studies of AN, the main outcome is the proportion of trials in which the individual chooses a high-fat item over the reference item. Two studies have demonstrated that this task captures the decreased selection of high-fat foods by individuals with AN, and task validity was additionally demonstrated by showing that proportion of high-fat choices correlated with actual caloric intake [60, 61]. This task can be used to examine processes contributing to maladaptive dietary restriction in AN.

#### Summary

Quality Index scores were most commonly affected by methods of recruitment, where the checklist is very conservative. The checklist requires studies to report enrolment either of the entire population or of consecutive patients, and a description of the source of recruitment for healthy individuals. The studies we included often did not meet these criteria for reporting, but nonetheless generally enrolled reasonable samples of individuals with AN. Some studies that did include large sample sizes also included individuals who did not meet full criteria for AN, which may limit the inferences that can be drawn [18, 24, 56]. Only one study included a power calculation [26]. Often sample sizes were small (e.g. [19, 52, 53, 62]). These factors were considered in the summary of findings.

Neuropsychological studies have captured differences in the cognitive appraisal of food in AN, and these findings have informed theoretical models of AN. Specifically, findings from reward processing tasks support the hypothesis that abnormalities in neural reward systems underlie the development and maintenance of AN [7, 63]. Differences in attention towards food between AN and HC have not been consistent; whether attention relates to eating in AN is worthy of further investigation. Decision-making (food-choice) paradigms capture the characteristic eating behaviour of individuals with AN. These paradigms, if studied in connection with actual food intake, may yield insights into mechanisms of AN.

### Neurophysiology

Physiological changes can index attitudes that are outside of conscious awareness [64]. Interest in measuring physiological responses to food in AN stems from clinical observations suggesting that individuals with AN may have difficulty describing symptoms and emotions.

#### Attention

Event related potentials (ERPs) are fluctuations in the brain’s electrical activity, or electrical potentials, that are measured via scalp electrode electroencephalography (EEG). EEG has high temporal resolution, allowing for separation of ERPs into different components [65]. An ERP recording during stimulus presentation contains distinct components of early orienting and sustained attention [66]. Three studies measured ERPs during presentation of food images (Table 2, Event-related potential). Two studies demonstrated that when food stimuli (but not neutral stimuli) were presented, individuals with AN initially display heightened ERP amplitudes compared to HC [67, 68]. At later stages of stimulus presentation, heightened ERP amplitudes in response to low-calorie foods in AN has been reported [68], although another study found no group difference in response to food versus non-food stimuli [69]. A related technique, magnetoencephalography (MEG), measures the electromagnetic field generated by the electrical activity of neurons, indexing brain activity also with high temporal resolution [70]. The one study using this technique reported heightened early activity in response to food stimuli in individuals with AN relative to HC [33].

#### Reward

Electromyography (EMG) tasks involve the recording of facial muscle contractions in response to images of food (Table 2, Electromyography). The amplitude of zygomatic (smiling) and corrugator (frowning) muscles are positively and negatively associated with pleasantness evaluations, respectively. When food images were presented following emotional primes (faces displaying happy, fear, disgust and neutral expressions) zygomatic responses to food images were decreased in individuals with AN, relative to HC [71]. A second study that included neutral stimuli found reduced zygomatic activity in AN versus HC for both types of images [72]. There was some indication of greater corrugator activity specifically in response to food stimuli in AN in both studies, but this varied with state (fasted or sated) and emotional priming. These studies also measured skin conductance and heart-rate changes; differences specific to food stimuli were not observed. The only EMG study of the levator labii muscle, associated with disgust, found activity increased among AN versus HC when participants viewed cues associated with chocolate [73]. This study also found increased zygomatic activity among AN in response to cues predicting the absence of chocolate. EMG has probed the startle eye-blink response to food, by measuring contraction of the orbicularis muscle that indexes aversion. Two studies found no startle-eye blink differences between AN and HC in response to food stimuli [74, 75]. Another reported that individuals with AN had difficulties reducing their startle eye-blink responses towards food stimuli, as well as negative emotional stimuli, though there was no comparison group [76].

#### Summary

Quality Index scores were affected by the failure to fully report method of recruitment or failure to include exact p-values (e.g. [73]). Only one study was limited by the lack of a comparison group [76]; some studies had the added strength of comparing acutely ill patients with recovered individuals in addition to healthy individuals [33, 75]. A selection of studies had the added rigor of either standardizing or controlling for states of feeding/hunger [33, 69, 71, 72, 74, 75].

Neurophysiological approaches are less developed than neuropsychological, and may not have reached their full potential. ERP and MEG evidence suggest greater initial attention to food among individuals with AN, though sustained attention findings are inconsistent. EMG findings suggest a reduction in hedonic response to food. These physiological assessments provide another angle to examine neural mechanisms underlying responses to food.

### Neuroimaging (Functional Magnetic Resonance Imaging, fMRI)

Neural correlates of illness have been examined using fMRI. One approach elicits psychiatric symptoms by presenting disorder-relevant stimuli, and measures associated neural activity (symptom provocation). Another method administers a task with known neural correlates to probe the functioning of the relevant brain system (cognitive activation probe; [77]). Food-image paradigms have been used to develop and test models of the neuropathology underlying AN.

#### Passive Viewing of Food

In passive viewing studies participants are asked to observe food stimuli during fMRI scanning (symptom provocation; Table 2, Passive viewing). Three studies reported altered amygdala activity among individuals with AN compared with HC [44, 45, 78], and three studies reported altered insula activation [45, 47, 78], however the direction of differences was not consistent between studies. Two studies reported alterations within visual processing areas in AN when high-calorie food was viewed, although exact loci and direction of difference was inconsistent [48, 79]. One study did not find differences in neural activity between AN and HC, but did report that neural activity during food-image viewing was differentially associated with stomach sensation ratings measured outside of the scanner in AN vs HC [80]. Collectively passive viewing studies have produced a diverse set of findings that are difficult to interpret.

#### Directed Food Tasks

To constrain the psychological experience when viewing food images (and in turn constrain the interpretation of findings), participants are given an instruction for how to internally process the images (symptom provocation, Table 2, Directed food tasks). When participants were asked to imagine eating presented foods, one study reported increased middle frontal gyrus activation in AN relative to HC [49], and another reported increased dorsolateral prefrontal cortex activity in AN [81]. When participants were asked to think about how hungry the presented foods made them, individuals with AN displayed greater ventromedial prefrontal cortex activity compared with HC [50]. When participants were instructed to think about how much they wished to eat each food item, increased frontal pole activity for high calorie foods was reported in AN but not HC [43]. A common finding amongst instructed viewing studies was altered frontal cortex activation in AN versus HC, though specific regions differed.

#### Active Food Choice

The food choice task (described in Neuropsychology) [60, 61] provides a cognitive activation probe, in that it assesses active decision-making and the neural correlates in HC are known [59]. FMRI data showed that food choice was associated with dorsal striatum activity among individuals with AN, but not HC [61]. The dorsal striatum and dorsolateral prefrontal cortex were functionally correlated, and this connectivity was related to intake among individuals with AN. By linking the task with actual eating behaviour, this study provides compelling data that dorsal frontostriatal circuit activity is associated with the maladaptive food choices observed in AN.

#### Summary

Quality Index scores were again affected by recruitment methods or descriptions (e.g. [79, 80, 82]). The majority of the studies involved very small numbers of participants, by current standards for fMRI investigations. Conclusions may be more robust for investigations with sample sizes greater than 20 per group (e.g. [61, 80, 83]). Some studies have the additional methodological strength of standardizing time since meals [43, 45, 47–50, 61, 79, 81, 84, 85], and of including non-food stimulus comparisons [44, 45, 47–50, 79–85].

Neuroimaging studies involving passive viewing of food have not identified consistent group differences. Adding an instruction for viewing has identified differences in frontal regions, in varying locations. Neural regions have multiple functions; various different processes may be captured during the viewing of food stimuli (even with instructions), making it difficult to draw inferences about mechanisms of illness from these paradigms. The only approach that linked neural activity with eating behaviour identified dorsal frontostriatal circuit engagement in AN. This is a promising finding that, if replicated, can open new avenues for research [86].

### Discussion

Tasks using images of food have been administered in 50 different studies of AN, using neuropsychological, neurophysiological, and neuroimaging techniques. This systematic review primarily assessed the utility of the experimental approaches. Nonetheless, some conclusions can be drawn from the data. First, while attention is related to intake among healthy individuals [17], data from four studies suggest that attention and eating behaviour are dissociated among individuals with AN [23, 25–27]. While a small collection of neurophysiological evidence suggests greater attentional orienting towards food stimuli in AN as compared to HC, findings from neuropsychological studies of attention are inconsistent. Second, measures of liking, value, and choice from neuropsychological and neurophysiological studies converge to indicate that individuals with AN have a decreased preference for high-fat/calorie foods. Third, while it is one study, the only data that linked maladaptive eating with neuroimaging indicated that this behaviour is associated with dorsal frontostriatal activity [61] .

The paradigms and approaches that have been used to date have some notable strengths, as well as important weaknesses. The strengths of the neuropsychological tasks are that they have probed constructs of interest, and some have well characterized underlying neural mechanisms. For example, wanting and liking tasks have been associated with mesolimbic activity among HC, suggesting they probe reward systems [87, 88]. The strengths of the neurophysiological measures include the objectivity of the assessment, as well as temporal resolution that offers a granular study of cognitive processes (i.e., assessing components of broad cognitive processes) [65, 89]. Neuroimaging methods do not have the same temporal resolution, but do have spatial resolution that enables identification of relevant brain regions [90]. Development of paradigms within these fields may allow the parsing of distinct neurocognitive processes and circuits, for an understanding of their role in AN.

The majority of studies involved small sample sizes and specific populations that limit generalizability. The studies ranged in quality, as assessed by the checklist. While this index is the most appropriate available, it is not specifically designed for cognitive neuroscience studies and so the index score does not address the specificity of the data collected. For example, neuropsychological tasks engage multiple neurocognitive domains, even when one variable is identified as the primary outcome. Techniques like EMG and EEG have limited spatial resolution, making neurologic interpretation difficult [65, 91]. FMRI data also captures multiple processes - particularly when responses are not constrained. It is a limitation of this systematic review that the assessment of each paradigm may be influenced by the quality of the study.

The major limitation across paradigms has been the absence of information about the relationship between task variables and eating behaviour among individuals with AN. One interesting dilemma in this research is how best to utilize cognitive neuroscience when the behaviour of interest differs between groups. That is, for many of these tasks, the association between task variables and eating behaviour has been established in HC, but this relationship is not present in AN. For example, ‘liking’ and ‘wanting’ are associated with actual eating among individuals without an eating disorder [87, 88], and attention is associated with food intake among HC [17]. Yet, the association between attention and intake is not so clear in AN, as shown in the studies reviewed here. On the one hand, it is interesting and potentially important to learn that these processes are dissociated in AN. On the other hand, interpreting the finding requires additional information about the relationship between attention and the maladaptive eating behaviour characteristic of AN.

Approaches used to study eating behaviour in populations of individuals without an eating disorder may offer new ideas that can enhance the utility of existing tasks in AN. By studying the effects of experimental manipulations on food choice, causal influences on eating behaviour have been identified in healthy individuals. For example, the role of attention to particular characteristics of food was assessed by asking participants to focus specifically on one value (tastiness or healthiness) of presented foods when making choices. Receiving the instruction to focus on healthiness increased the influence of healthiness on decisions. FMRI data showed that the change in preference was associated with greater functional connectivity between the dorsolateral and ventromedial prefrontal cortices [92]. This cognitive manipulation may be useful in understanding decision-making processes in AN, again highlighting the utility of food choice paradigms.

In motor approach training, participants learn to press a button in response to particular food images presented on-screen. The comparison of food value ratings made before and after the training demonstrated that the approach training increased the reward value of the associated foods and increased choice of these foods [93]. On the flip side, with motor inhibition training individuals learn to inhibit or withhold automatic approach responses to food stimuli. Among overweight and obese participants, this resulted in decreased liking of high-calorie foods, reduced neural activity in reward-signalling regions in response to high-calorie foods, and reduced caloric intake [94–97]. Motor approach or inhibition may be useful in AN, as well, where changing value assessment and choice of foods may be therapeutic [98].

Three ideas emerge from this review that may advance research. One, the link between experimental measurements and the eating behaviour characteristic of AN is critical to establishing mechanisms of illness. Two, introducing experimental manipulations may allow for inferences about causality. Neuroimaging in conjunction with manipulations may further elucidate the neural underpinnings of maladaptive eating. Three, perhaps most importantly, clear hypotheses should guide the selection of tasks and approaches, so that research questions may be fully addressed, and in a reliable and valid manner. Much of the food-image research to date has been descriptive; it is time to begin testing the mechanistic hypotheses that have emerged.

## Abbreviations

AN: Anorexia nervosa
EEG: Electroencephalography
EMG: Electromyography
ERP: Event-related potential
FMRI: Functional magnetic resonance imaging
MEG: Magnetoencephalography
HC: Healthy controls
